# Ultrasensitive and label free electrochemical immunosensor for detection of ROR1 as an oncofetal biomarker using gold nanoparticles assisted LDH/rGO nanocomposite

**DOI:** 10.1038/s41598-021-94380-5

**Published:** 2021-07-21

**Authors:** Rozita Abolhasan, Balal Khalilzadeh, Hadi Yousefi, Sahar Samemaleki, Forough Chakari-Khiavi, Farzaneh Ghorbani, Ramin Pourakbari, Amin Kamrani, Alireza Khataee, Tannaz Sadeghi Rad, Mohammad Reza Rashidi, Mehdi Yousefi, Leili AghebatiMaleki

**Affiliations:** 1 Stem Cell Research Center (SCRC), Tabriz University of Medical Sciences, 51664-14766 Tabriz, Iran; 2 Student Research Committee, Tabriz University of Medical Sciences, Tabriz, Iran; 3 Biosensor Sciences and Technologies Research Center, Ardabil University of Medical Sciences, Ardabil, Iran; 4 Department of Basic Medical Sciences, Khoy University of Medical Sciences, Khoy, Iran; 5 Student Research Committee, Hamadan University of Medical Sciences, Hamadan, Iran; 6 Pharmaceutical Chemistry, Faculty of Pharmacy, Tabriz University of Medical Sciences, Tabriz, Iran; 7 Research Laboratory of Advanced Water and Wastewater Treatment Processes, Department of Applied Chemistry, Faculty of Chemistry, University of Tabriz, Tabriz, Iran; 8 Department of Environmental Engineering, Gebze Technical University, 41400 Gebze, Turkey; 9 Department of Materrial Science and Physical Chemistry of Materials, South Ural State University, 454080 Chelyabinsk, Russian Federation; 10 Research Center for Pharmaceutical Nanotechnology, Tabriz University of Medical Sciences, Tabriz, Iran; 11 Aging Research Institute, Tabriz University of Medical Sciences, Tabriz, Iran; 12 Department of Immunology, Faculty of Medical Sciences, Tabriz University of Medical Sciences, Tabriz, Iran; 13 Immunology Research Center, Tabriz University of Medical Sciences, PO Box 6446-14155, Tabriz, Iran

**Keywords:** Cancer, Biomarkers, Medical research, Chemistry

## Abstract

In the present article, we developed a highly sensitive label-free electrochemical immunosensor based on NiFe-layered double hydroxides (LDH)/reduced graphene oxide (rGO)/gold nanoparticles modified glassy carbon electrode for the determination of receptor tyrosine kinase-like orphan receptor (ROR)-1. In this electrochemical immunoassay platform, NiFe-LDH/rGO was used due to great electron mobility, high specific surface area and flexible structures, while Au nanoparticles were prepared and coated on the modified electrodes to improve the detection sensitivity and ROR1 antibody immobilizing (ROR1Ab). The modification procedure was approved by using cyclic voltammetry and differential pulse voltammetry based on the response of peak current to the step by step modifications. Under optimum conditions, the experimental results showed that the immunosensor revealed a sensitive response to ROR1 in the range of 0.01–1 pg mL^−1^, and with a lower limit of quantification of 10 attogram/mL (10 ag mL^−1^). Furthermore, the designed immunosensor was applied for the analysis of ROR1 in several serum samples of chronic lymphocytic leukemia suffering patients with acceptable results, and it also exhibited good selectivity, reproducibility and stability.

## Introduction

The receptor tyrosine kinase-like orphan receptor 1 (ROR1), an oncofetal antigen, is a Wnt5a receptor and is widely expressed during embryogenesis, whereas it is absent in most adult tissues^1,2^. Studies have reported that ROR1 is expressed at a high level in human leukemia and several solid malignancies^3–5^, suggesting that detection of ROR1 as a prognostic biomarker is important for the on time clinical analysis and the long-term treatment monitoring. Up to now, numerous conventional techniques have been used for the evaluation of ROR1, including flow cytometry^6^, enzyme-linked immunosorbent assay^7^, western blotting^8^, immunohistochemistry^9^, and polymerase chain reaction^10^, but most of the above-mentioned methods have several limitations, such as expensive instruments, complication, label-required, time-consuming, and inappropriate for point-of-care diagnosis. Thus, there is a real need for the development of a simple, rapid, sensitive and inexpensive method for evaluation of ROR1 biomarker. Electrochemical immunosensors are based on the Ag–Ab complexes formation serve as a suitable method with high-sensitivity, specificity and have gained much attention in clinical diagnosis due to their advantages of fast response time, simple operation, high efficiency and low cost^11–13^.

To design of ultra-sensitive immunosensors, signal amplification and antibody immobilization steps are very essential^14–18^. Therefore, choosing the ideal material to reach this goal is important in developing a high-performance biomarker detection platform. Nanomaterials have been extensively used for the design and fabrication of electrochemical sensors and immunosensors^19,20^. Also, because of their profound advances such as high surface area, excellent conductivity, outstanding biocompatibility, high receptor loading efficacy^21–23^. Layered double hydroxides (LDHs), as a class of lamellar nano-compounds, have attracted considerable interest due to high specific surface, flexible structures and ion-exchange ability^24^. The key advantages of these 2D structures are that they can be fabricated by the different metal cations and the exchangeable intercalated anions^25^. Nevertheless, the poor conductivity of LDH often limits electron transfer; so, their electrochemical activity can be enhanced excellently when they are combined with nano-carbon materials^26^. Therein, carbon materials such as reduced graphene oxide (rGO) can avoid the LDH agglomeration; improve the electrochemical activity, and leads to high-rate performance and better cycling stability for the preferred devices^27^. Also, graphene has peculiar advantages as well as high surface area, distinctive electronic properties, great thermal and chemical stability, and excellent mechanical flexibility^28,29^.

Studies showed that gold nanoparticles (AuNPs) are sort of renowned metal-nanomaterials that have been broadly used for immobilizing antibodies due to their excellent biocompatibility and easy immobilization of bioreceptors (antibody, aptamer and RNA/DNA) on themselves^30–32^ and also the electrical conductivity of the electrochemical platforms could be significantly improved^33,34^. As a result, NiFe-LDH/rGO/AuNPs nanocomposite can be used as an exceptional electrochemical platform for the fabrication of immunosensor, because of the combination of physical and chemical advantages of NiFe-LDH/rGO and the more active sites of AuNPs for capturing a large amount of antibodies.

In the present study, a new label-free electrochemical platform based on NiFe-LDH/rGO nanocomposites and AuNPs was designed and constructed for the detection of ROR1 biomarker. A stable NiFe-LDH/rGO nanocomposite with appropriate and acceptable electrocatalytic activity was first electrodeposited on the glassy carbon electrode (GCE). Then, AuNPs were coated onto the modified electrode by electrodeposition, which used as the mediator for ROR1 Ab capturing and acted as signal amplifiers for the immunosensor. Electrochemical detection for ROR1 was done using differential pulse voltammetry (DPV) in the K_4_[Fe(CN)_6_] solution. The developed immunosensor presents great performance and showed high sensitivity and strange specificity. Furthermore, the proposed sensing method can be employed to detect ROR1 in human serum samples with acceptable results, which provides an effective strategy in clinical research. Label free electrochemical immunosensor with high sensitivity can detect very low amount of ROR1 and thus help in the point of care diagnosis of most cancers. It will also be more cost-effective and simpler than conventional ROR1 detection methods. In addition, the label free methods are more time consuming ones in comparison with labeled methods. In other word the incubation time of secondary antibody with the target protein is neglected.

## Methods

### Reagents and apparatus

Chloroauric acid (HAuCl_4_) was purchased from Shiraz Chemical Company (Shiraz, Iran). Polyethylene glycol 6000 was purchased from Sigma–Aldrich (USA). Potassium ferricyanide K_4_[Fe(CN)_6_] was purchased from Aladdin Company (Shanghai, China). Ethanol (C_2_H_6_O), sulfuric acid (H_2_SO_4_), nitric acid (HNO_3_) and citric acid (C_6_H_8_O_7_) were purchased from Merck (Darmstadt, Germany). The receptor tyrosine kinase-like orphan receptor (ROR)-1 was obtained from R&D (Minneapolis, MN, USA). ROR1 ELISA kit purchased from Raybiotech Company (USA). Distilled water was used in all the experiments.

All electrochemical measurements, including differential pulse voltammetry (DPV) and cyclic voltammetry (CV), were done with Autolab potentiostat/galvanostat (Metrohm) and for data processing controlled with NOVA 1.8 software. A conventional three-electrode system was employed for all electrochemical measurements with a platinum wire as the counter electrode, Ag/AgCl (with 3 M KCl) as the reference electrode and a modified 2-mm-diameter glassy carbon electrode (GCE) as the working electrode; glassy carbon electrode was bought from Azar Electrode Company (Urmia, Iran). The scanning electron microscopy (SEM) images of electrodeposited NiFe-LDH/rGO and AuNPs on GC electrode were taken on a VEGA TESCAN, Czech Republic apparatus. Initially, at the vacuum condition, the samples were exposed to electron bombardment of gold atoms, then for taking images the samples were moved to SEM chamber.

### Preparation of NiFe-LDH/rGO and AuNPs solution

The NiFe-LDH/rGO nanocomposite was synthesized by a hydrothermal method following a previously described in Khataee et al. study^24^. At first, a solution of 3 mmol Ni(NO_3_)_2_ and 1 mmol Fe(NO_3_)_3_ was mixed and added to 2 mol/L NaOH solution at 25 °C and stirred for 30 min until reached to the pH of 9. Next, the solution was mixed with 0.02 g GO that dissolved in 10 mL of ultrapure water and stirred for 30 min. In the next step, the solution was transferred into a pre-heated oven at 110 °C for 19 h, the gained precipitate was washed with distilled water and dehydrated in the oven at 60 °C for 18 h. To preparation of NiFe-LDH/rGO solution for electrodeposition onto GCE, 0.01 g of NiFe-LDH/rGO powder was dispersed in 1 mL distilled water and sonicated at least for 30 min. Then, 600 µL of homogenized NiFe-LDH/rGO solution was mixed with 4.4 mL of KNO_3_ (0.1 M). Before used for electrodeposition, this solution was sonicated for 15 min each time. To preparation of AuNPs solution for electrodeposition, 0.03 g/mL HAuCl_4_.3H_2_O was diluted with 15 ml distilled water. Then, 5 ml of prepared Au solution (0.01 g/ml) was mixed with 0.05 g of KNO_3_ (0.1 M).

### Fabrication of the BSA/Ab/AuNPs/NiFe-LDH/rGO/GCE immunosensor

**Scheme 1:**
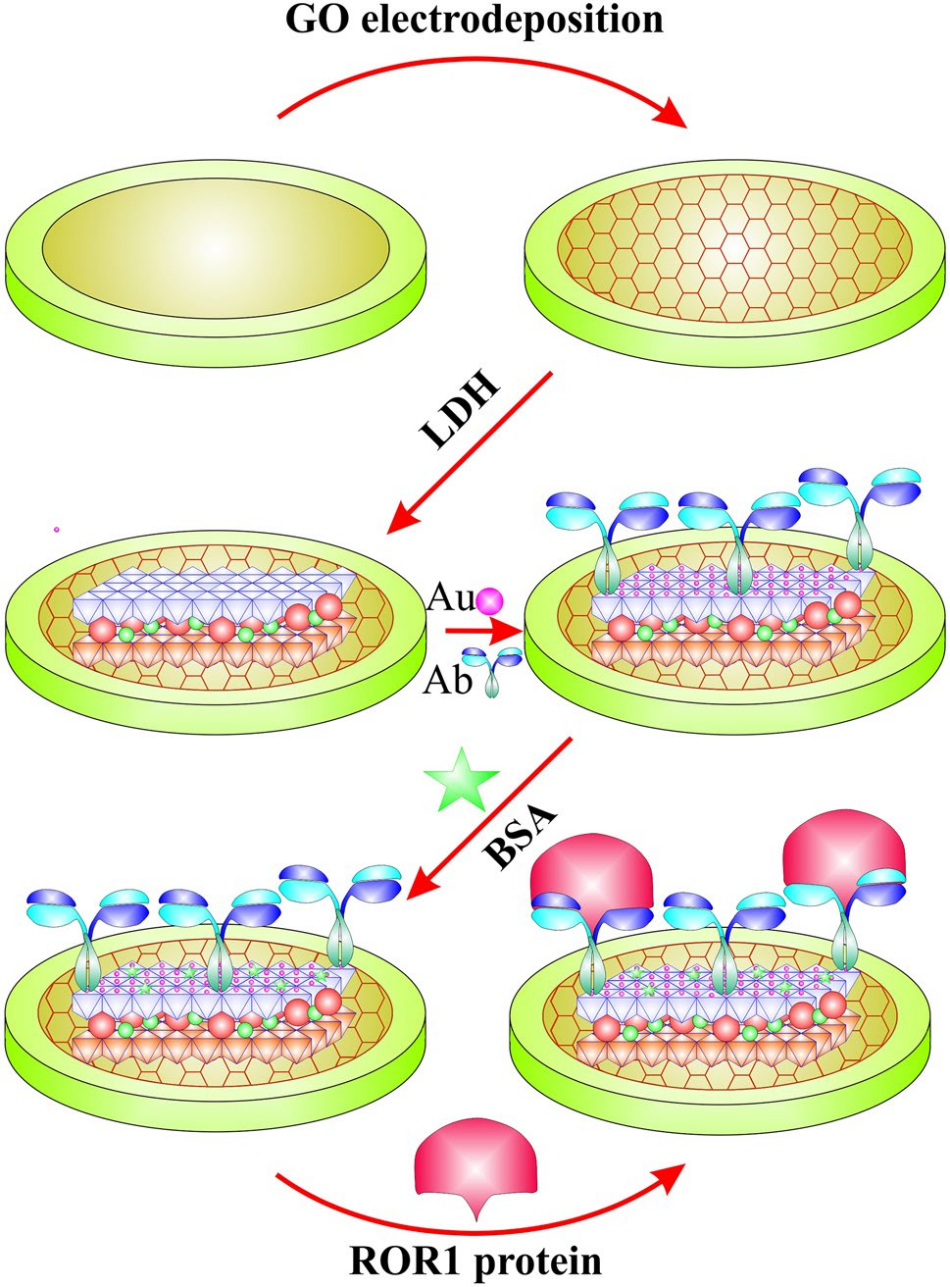
Schematically presentation of Electrode preparation steps.

Scheme 1 shows the stepwise procedure for the fabrication of the electrochemical immunosensor. Before use, the GCE was polished repeatedly in 0.3 μm and 0.05 μm alumina powder to obtain a mirror-like surface. Then, the electrode was sequentially sonicated in distilled water and ethanol, each for 5 min, and dried with nitrogen gas. Subsequently, the electrode was cleaned electrochemically in 0.5 M H_2_SO_4_ aqueous solution under a cycling electrode potential from − 0.3 to 1.55 V with a scan rate of 100 mV s^-1^. The pre-treated GCE was immersed in NiFe-LDH/rGO aqueous solution and kept at a cycling potential of − 0.6 to 1.5 V for 10 cycles with a scan rate of 50 mV s^−1^ to electrodeposition of NiFe-LDH/rGO on the surface of GCE. The obtained electrode was washed with distilled water, permitted to dry under room temperature and represented as NiFe-LDH/rGO/GCE. Afterward, the NiFe-LDH/rGO/GCE was immersed in 0.01 M HAuCl_4_.3H_2_O solution and electrochemical synthesis of AuNPs was carried out on the surface of the NiFe-LDH/rGO/GC electrode by cyclic voltammetry in the potential range from − 0.5 to 0 V for 25 cycles with the scan rate of 50 mV/s. After rinsed with ultrapure water, the AuNPs/NiFe-LDH/rGO/GCE was gained. To preparation of Ab/AuNPs/NiFe-LDH/rGO/GCE immunosensor, 10 µL of monoclonal Ab of ROR1 at the concentration of 15 µg/mL in 0.1 M phosphate buffer (pH 7.3) was dropped onto the AuNPs/NiFe-LDH/rGO/GCE surface and incubated overnight at 4 °C, followed by washing with PBS buffer. Then, the Ab/AuNPs/NiFe-LDH/rGO/GCE was cast with 1% BSA solution and incubated at 37 °C for 1 h to block the remaining active sites and prevent nonspecific binding between ROR1 protein and AuNPs/NiFe-LDH/rGO/GCE. After washing with ultrapure water, the obtained electrode was characterized as BSA/Ab/AuNPs/NiFe-LDH/rGO/GC electrode. All immobilization stages were checked and approved by the electrochemical techniques (CV and DPV) which were performed in a standard solution of K_4_[Fe(CN)_6_] (3 mL, 5 mmol·L^−1^) containing 0.1 M KCl.

### Electrochemical assay of ROR1

The BSA/Ab/AuNPs/NiFe-LDH/rGO/GCE was incubated with 10 µL aqueous solution containing different concentrations of ROR1 protein for 2 h at 25 °C, followed by washing with distilled water. Then, the final modified electrode (ROR1/BSA/Ab/AuNPs/NiFe-LDH/rGO/GCE) was examined by DPV technique in 10 mL of 5 mM K_4_[Fe(CN)_6_] solution (pH 7.0) in the potential range from − 0.1 to 0.6 V with scan rate of 100 mV/s. Each electrochemical measurement was repeated at least three times. The control tests for cycle number of electrodeposition of NiFe-LDH/rGO and AuNPs, incubation time of Ab and ROR1 protein were done at the equal experimental circumstances.

### Ethics approval and consent to participate

All patients were asked to complete the informed consent. All procedures of this study was approved by Local Ethics Committee of Tabriz University of Medical Sciences (IR.TBZMED.VCR.REC.1397.179). All procedures were done in accordance with the declaration of Helsinki. Volunteers were asked to complete informed consent form before participation into the study.

### Consent for publication

Not applicable.

## Results and discussion

### Choice of materials

Nanomaterials, as flexible and stretchable devices, which display tremendous sensitivity and stability can be applied to electrochemical immunosensors^35,36^. To get high performance, the following nanomaterials were carefully chosen for immunosensor structure. We have used the NiFe-LDH/rGO nanocomposite in the fabrication of immunosensor for the first time, by considering that has high conductivity to increase the electron transfer, high available active sites, higher ratio of surface to volume, high specific capacitance, great dispersion, and as a low-cost electrocatalyst.

Also, on this fabricated immunosensor, gold nanoparticles (AuNPs) were adopted as effective nanomaterial for oriented immobilization of bioreceptors. AuNPs on the modified electrode can not only increase the amount of immobilized antibodies, but also speed up the electron transfer rate for signal amplification and thus increases the sensitivity of the designed immunosensor^37,38^. Therefore, the combination of NiFe-LDH/rGO nanocomposite and AuNPs should be a good immunosensing platform.

### Electrochemical characterization of the immunosensor

Scheme 1 displays the construction procedure of the electrochemical immunosensor. For the detection of ROR1, NiFe-LDH/rGO nanocomposites were synthesized by a hydrothermal method in the first stage. As showed in Scheme 1, a stable NiFe-LDH/rGO nanocomposite was coated on the GCE as an electrocatalyst agent. Thereafter, AuNPs were coated onto the modified electrode by electrodeposition, which was presented as a linker for ROR1 antibody.

**Figure 1:**
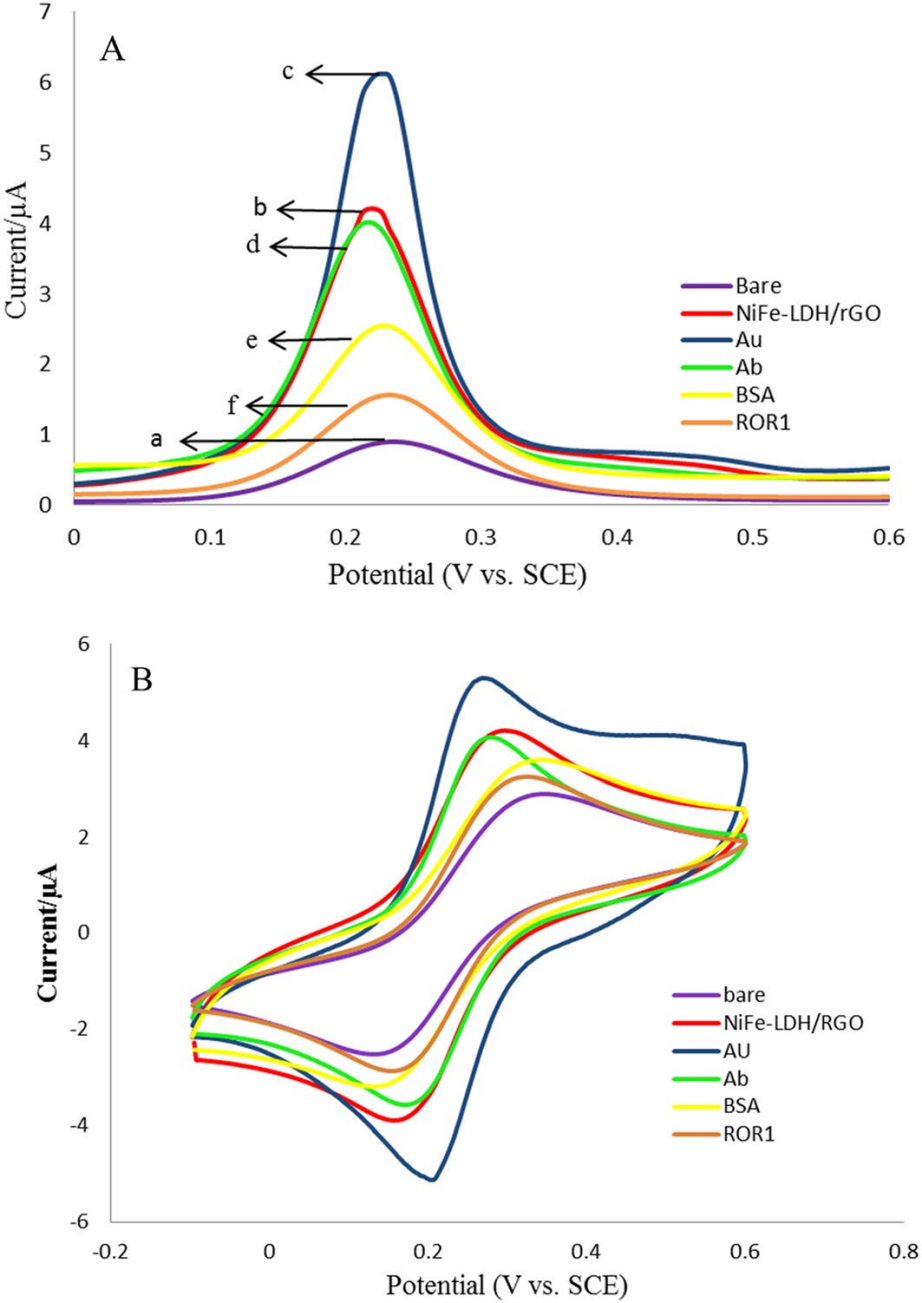
(**A**) The differential pulse voltammetry (DPV) of immobilization steps of the modified electrode in Fe(CN)63−/4−. Scan rate, 100 mV/s; (a) bare GCE, (b) NiFe-LDH/rGO, (c) NiFe-LDH/rGO/AuNPs, (d) NiFe-LDH/rGO/AuNPs/ROR1sAb, (e) NiFe-LDH/rGO/AuNPs/ROR1sAb/BSA, and (f) NiFe-LDH/rGO/AuNPs/ROR1sAb/BSA/ROR1sAg. (**B**) The cyclic voltammetry (CV) of immobilization steps of the modified electrode in Fe(CN)63−/4−. Scan rate, 100 mV/s.

The immobilization steps of the modified electrode were monitored by DPV and CV techniques. As shown in Fig. 1A in the DPV voltammogram of bare GCE, a noticeable oxidation peak was recorded at about 0.23 V (curve a). After the electrodeposition of NiFe-LDH/rGO nanocomposites on the bare electrode, the oxidation peak was obviously amplified, approximately four times of oxidation current of the bare electrode (curve b), which shows that the NiFe-LDH/rGO nanocomposites increased the electrocatalytic activity, electron transfer and conductivity. In the second step, HAuCl_4_.3H_2_O was electrochemically synthesized onto the NiFe-LDH/rGO nanocomposite modified electrode, so the oxidation peak was more increased about six times of oxidation current of bare electrode (curve c). This increase is due to AuNPs abilities in acceleration of the electron transfer rate and results in signal amplification. Also, AuNPs have used as the best interface to immobilization of bioreceptors i.e. antibodies on the modified electrode via Au-amine and Au-sulphur chemical bonds. Consequently, the stability of the developed immunosensor was significantly enhanced. Then, the oxidation peak current decreased after anti-ROR1 was immobilized onto the modified electrode (curve d); it indicates that ROR1 antibody was successfully immobilized on the modified electrode. Likewise, the peak current further declined after the electrode was blocked by BSA, as the non-electroactive substrate (curve e). Finally, after the incubation of the designed immunosensors with ROR1 protein, the oxidation peak was gradually decreased to a minimum because of the immunocomplex formation (curve f). Also, the CV (Fig. 1B) results approve the DPV results. Both the DPV and CV results support the successful fabrication of the immunosensor.

### Characterization of the modified GCE using SEM and EDS

**Figure 2:**
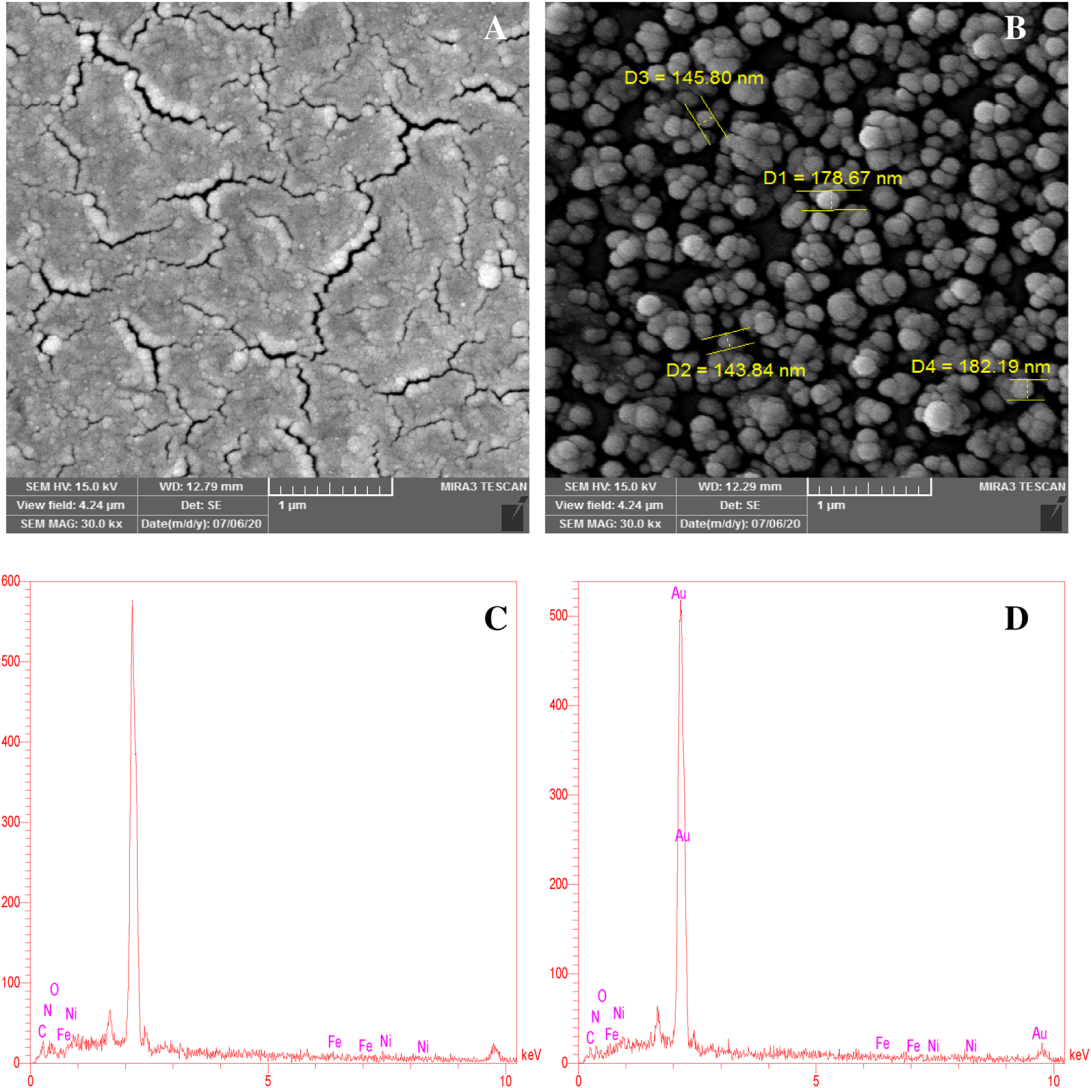
SEM images of NiFe-LDH/rGO nanocomposites (**A**); NiFe-LDH/rGO/AuNPs nanocomposites (**B**); EDS spectra of NiFe-LDH/rGO (**C**) and NiFe-LDH/rGO/AuNPs (D) on the GCE.

Figure 2A,B shows the scanning electron microscope (SEM) images of the NiFe-LDH/rGO as well as the NiFe-LDH/rGO/AuNPs modified GCEs. Figure 2A shows an irregular corrugated sheet-like structure, demonstrating the successful deposition of NiFe-LDH/rGO, and the frizzy surface can make a large active surface area for immobilizing of Au nanoparticles. In Fig. 2B, the SEM images show a large amount of spherical material, which showed that a large number of AuNPs were synthesized and immobilized onto the surface of the modified electrode and provide a large number of AuNPs modified surface for the immobilization of ROR1 Ab. The particle diameter of the AuNPs is about 140–200 nm. All of the electrode preparation steps were characterized by SEM and the images were illustrated in Supplementary Fig. S1.

Energy Dispersive Spectrometer (EDS) characterization as semi-quantitative method was used to analyze the chemical composition of NiFe-LDH/rGO nanocomposites. As shown in Fig. 2C, the fully scanned spectra demonstrated the characteristic elements of Ni (30.97%), Fe (15.19%), C (17.95%), N (23.04%) and O (12.86%), which confirms the formation of NiFe-LDH/rGO on the GCE. After the deposition of AuNPs on modified GCE, the signal related to Au could be observed (Fig. 2D), which are representing that AuNPs desirably coated onto the surface of the NiFe-LDH/rGO nanocomposites.

### Optimization of experimental conditions

To enhance the selectivity, sensitivity, and electrochemical signal of the immunosensor, the following experimental parameters were optimized: (a) the electrodeposition cycles number of NiFe-LDH/rGO; (b) the electrodeposition cycles number of AuNPs; (c) concentration of anti-ROR1.

**Figure 3:**
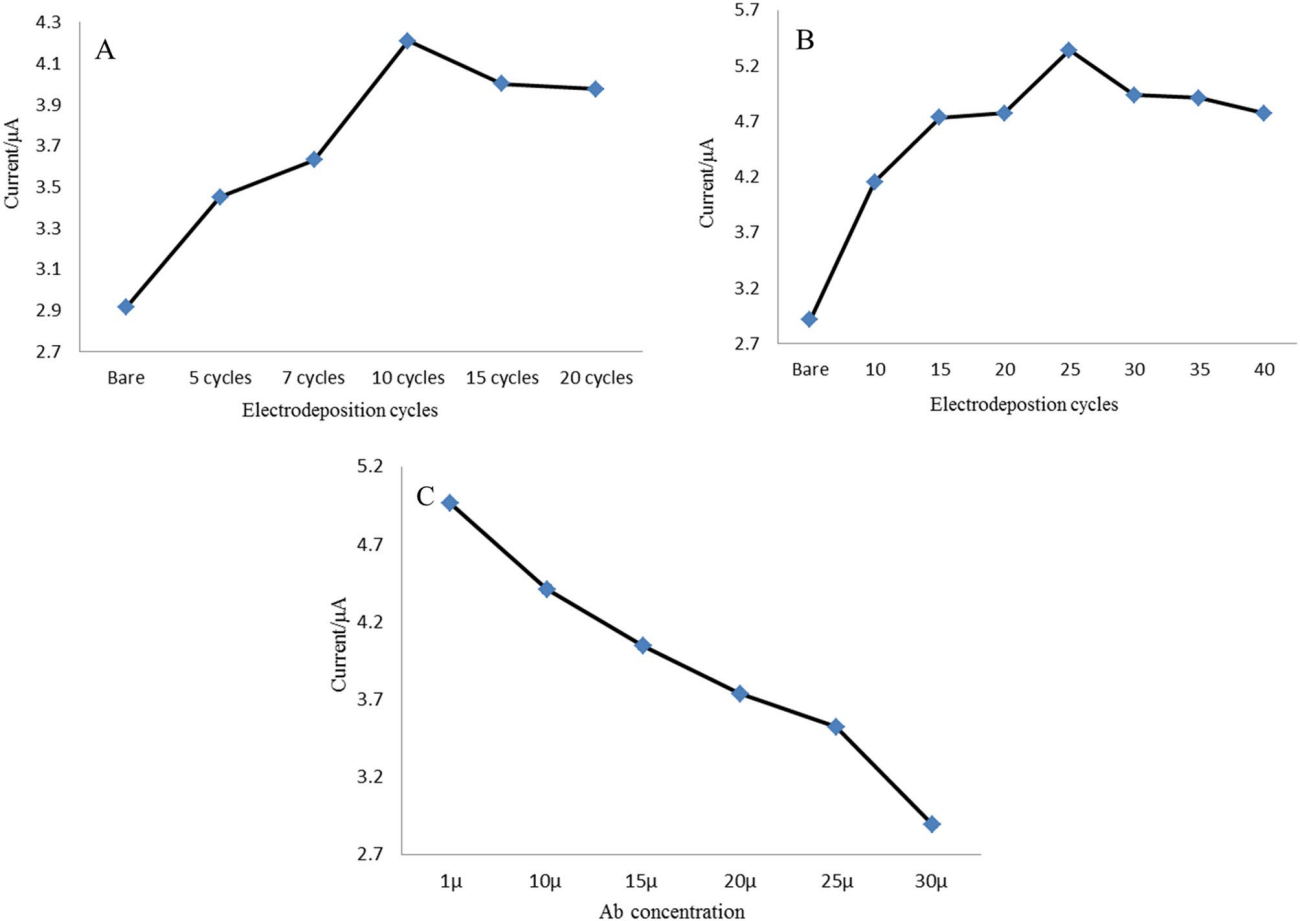
Optimization of the experimental conditions. Effect of (**A**) NiFe-LDH/rGO electrodeposition cycles number; (**B**) the AuNPs electrodeposition cycles number and (**C**) different anti-ROR1 concentration on the current response.

Figure 3A shows the result of the electrodeposition cycles number of NiFe-LDH/rGO on the oxidation peak current on the GCE after each electrodeposition step. The electrodeposition cycles number of NiFe-LDH/rGO was optimized with CV technique. Each cycles number was verified three cyclic voltammograms and the average of the oxidation peak currents were calculated and used. The peak current values increase with the increase of the electrodeposition cycles number of NiFe-LDH/rGO and a plateau reaches when the cycle number is 10. When the cycle number is more than 10, the value of NiFe-LDH/rGO decreases because of the increase in nanocomposite thickness and prevents the electron transfer. So, the electrodeposition cycle number of NiFe-LDH/rGO is chosen as 10 in the following assesses of ROR1.

AuNPs have been used as second layered in the construction of the immunosensor for immobilization of anti-ROR1. As recorded in Fig. 3B with increasing electrodeposition cycles number, the oxidation peak currents in CV technique, were increased until 25 cycles and then reached a plateau, which indicated an enhancement of electron transfer, so 25 cycles were selected as the optimal electrodeposition cycles number.

The concentration of the immobilized Ab is a critical factor in the fabrication of immunosensors in point of economic view. The effect of the various concentrations of Ab on the recorded peak current of the modified electrode, Ab/AuNPs/NiFe-LDH/rGO/GCE, was also examined and the conforming results are presented in Fig. 3C. The peak currents decreased with the increased ROR1Ab concentration, by increasing the Ab concentration and decreasing peak current too much leaves fewer limits for protein detection. Therefore, 15 µg/mL was chosen as the appropriate optimum concentration for ROR1Ab in the following tests.

### Ultra-sensitive performance of the immunosensor

**Figure 4:**
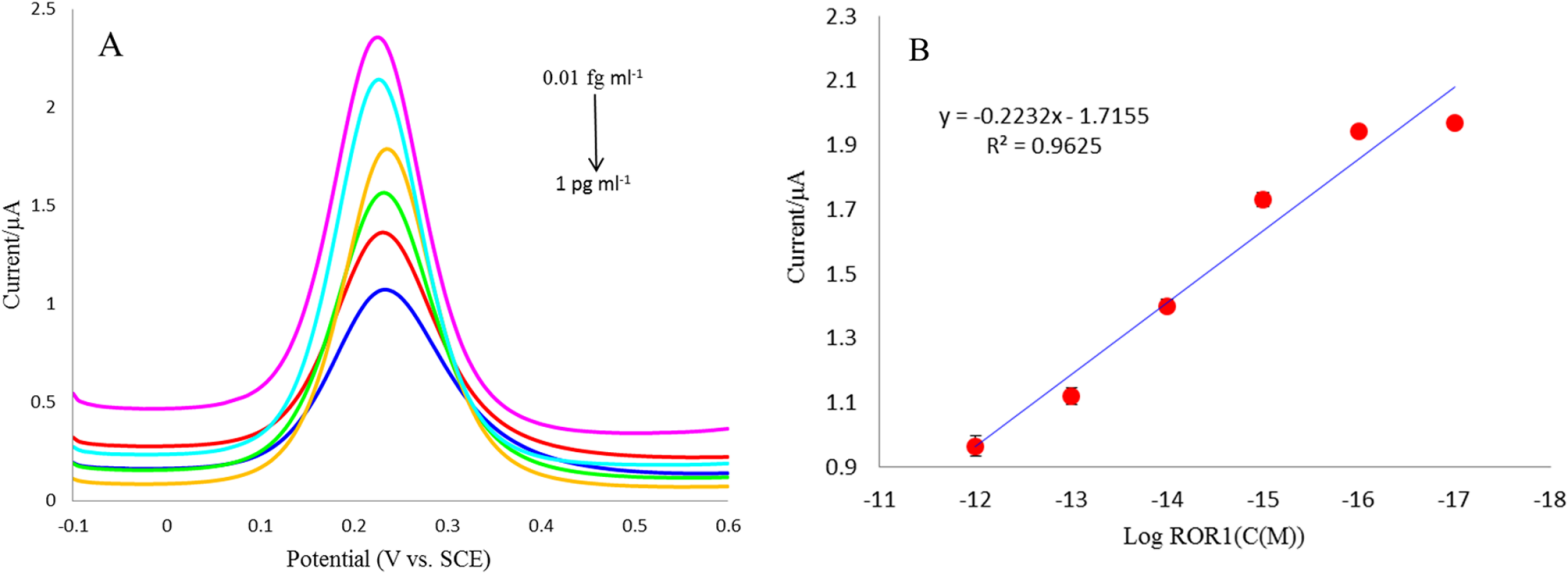
(**A**) DPV curves of the immunosensor after incubation with different concentrations of ROR1 (0.01 fg mL^−1^, 0.1 fg mL^−1^, 1 fg mL^−1^, 0.01 pg mL^−1^, 0.1 pg mL^−1^, 1 pg mL^−1^). (**B**) The linear relationship between the peak current versus the logarithm concentration of ROR1 in the range of 0.01 fg mL^−1^ to 1 pg mL^−1^. Error bars represent standard deviation (n = 3).

Under the optimal conditions, the sensitivity and quantitative range of the immunosensor were assessed by DPV responses to different concentrations of ROR1, at a usual working potential of − 0.1 to 0.6 V in 10 μL of K_4_[Fe(CN)_6_] solution at a scan rate of 100 mV s^−1^. As shown in Fig. 4A, by increasing the concentration of ROR1 protein in the range 0.01–1 pg mL^−1^ leads to the reduce of the peak current of DPV. The reason for this was that, by increasing the amount of immune-complex hindered the electron transfer on the modified electrode surface^39^. Figure 4B shows a wide linear relationship between the peak current and the logarithm values of the ROR1 concentrations. The regression equation is expressed as log I = − 0.2232 log C_ROR1_—1.7155 (R^2^ = 0.9625). As the sensitivity merit, the limit of detection (LOD) is estimated as 10 ag mL^−1^ (S/N = 3). The low detection limit and wider linear range of the suggested immunosensor should be related to the employment of NiFe-LDH/rGO/AuNPs with high conductivity and high active sites that increased the amount of immobilized Ab (as biorecognition element) that leading to enhance the chance of antibody–antigen immune-complex formation. Supplementary Table S1 presented the analytical performance of the current immunoassay has been compared with the performances of ELISA immunoassay for ROR1 detection, and the proposed immunosensor exhibited a low detection limit and an extensively linear dynamic range.

### Selectivity, reproducibility and stability of immunosensor

**Figure 5:**
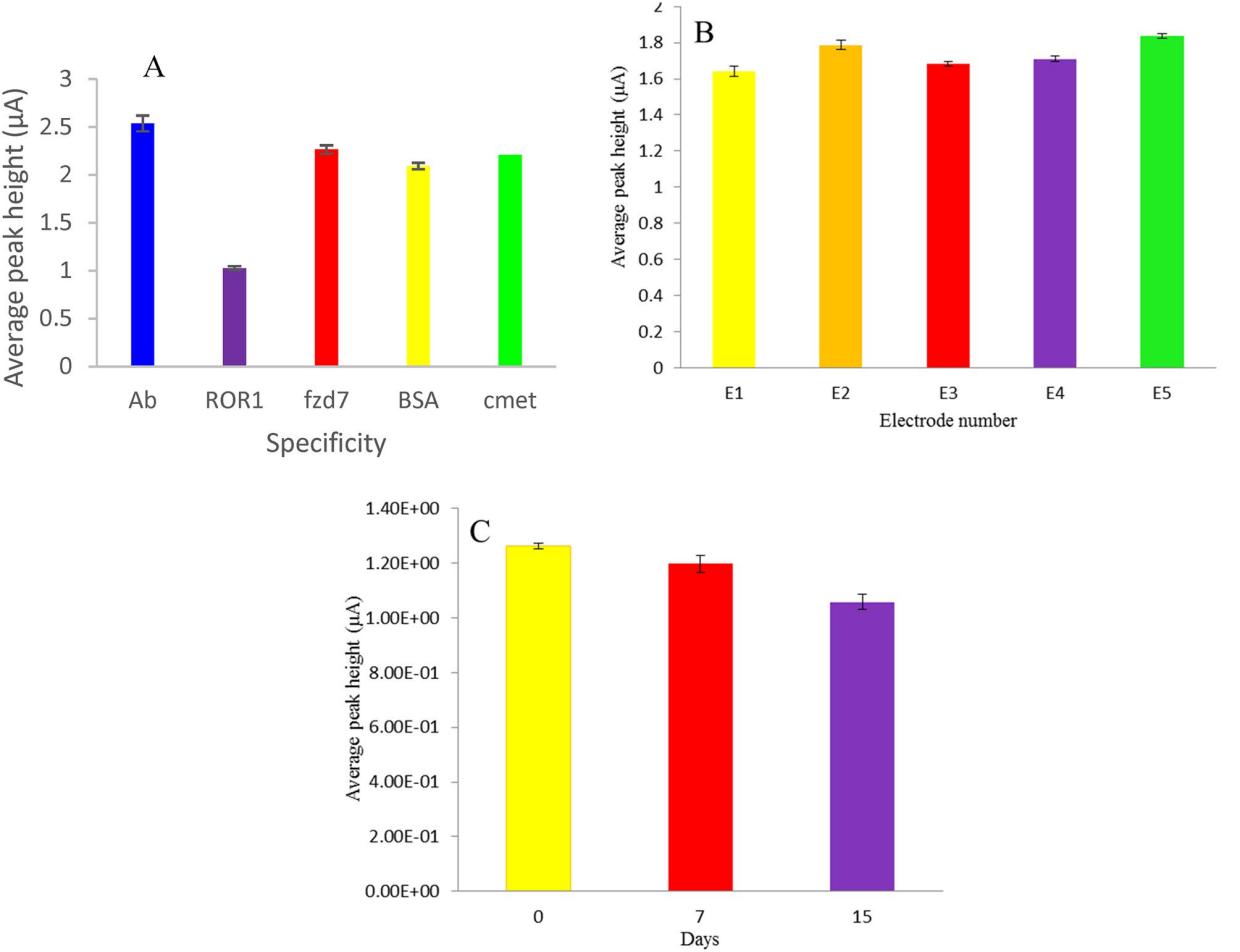
(**A**) Selectivity of immunosensor to ROR1 (1 pg mL^−1^) compared with four nonspecific interferences including cMet (1 pg ml^−1^), fzd7 (1 pg ml^−1^) and BSA (1 pg ml^−1^) (n = 3). (**B**) reproducibility of the immunosensor measured by analyzing the ROR1 with concentration of 1 fg ml^−1^ with five equal electrodes prepared by the same method. (**C**) stability of the ROR1 immunosensor kept at 7 and 15 days in refrigerator at 4 °C.

High specificity, reproducibility and stability of the recorded signals are essential for the performance of the immunosensor. To prove the specificity of the electrochemical immunosensor, the DPV voltammograms of the designed immunosensor to possible interferences such as cMet (1 pg mL^−1^), fzd7 (1 pg mL^−1^) and BSA (1 pg mL^−1^) were studied. Like ROR1, c-MET is a tyrosine kinase receptor and the cysteine-rich domain of fzd7 is shared in the ROR1 structure. As can be seen in Fig. 5A no significant change was observed in the signal responses of DPV to nonspecific interferes in comparison with achieve results in the presence of target protein, ROR1. These results show that the specificity of the developed electrochemical immunosensor was acceptable.

The reproducibility of the modified electrochemical immunosensor was also assessed with the inter-assay method. The inter-assay precision of the immunosensor was measured by analyzing the ROR1 with concentration of 1 fg mL^-1^ with five equal electrodes prepared by the same method the appropriate relative standard deviation (RSD) was obtained as 4.08% (Fig. 5B). These results indicating that the ROR1 immunosensor had a satisfactory precision and reproducibility.

Finally, the long-time stability of the BSA/Ab/AuNPs/NiFe-LDH/rGO/GC immunosensor was also studied by keeping the immunosensor in dry conditions at 4 °C. No significant change was observed in the current response after 15-days storage. The immunosensor remained over 5% and 16% of its initial response after 7 and 15 days’ storage, respectively, demonstrating good stability of the ROR1 assay (Fig. 5C).

### Real sample analysis of ROR1 biomarker

**Figure 6:**
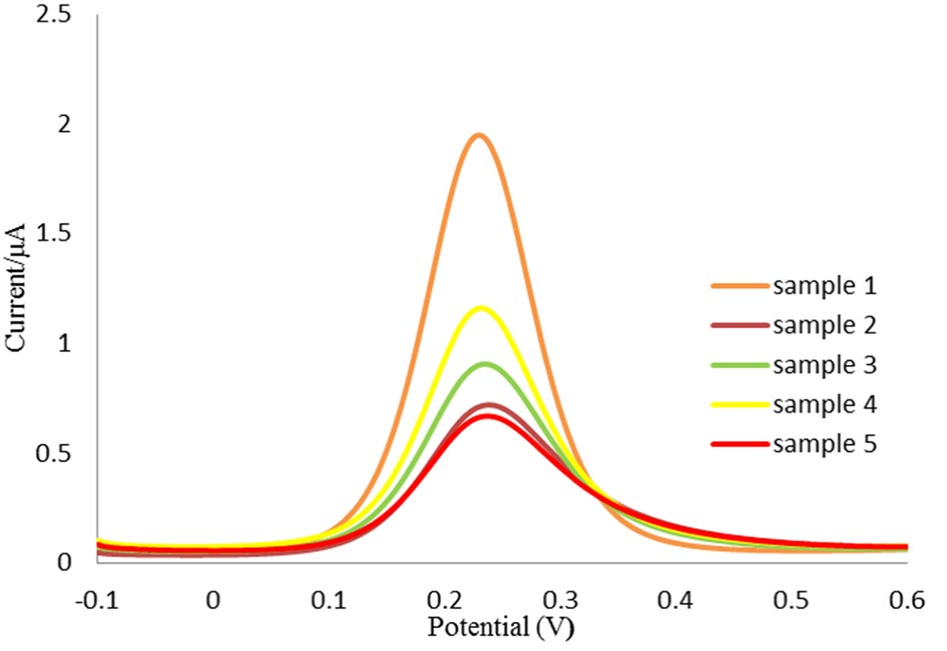
Real sample analysis of ROR1 biomarker in five clinical CLL human serum samples.

In order to evaluate the feasibility of the label-free electrochemical immunosensor, five clinical CLL human serum samples were measured. To this end, sera were collected from patients referred Shahid Ghazi hospital affiliated to Tabriz University of Medical Sciences. All patients were asked to complete the informed consent form. The content of ROR1 protein in the serum samples was determinate by the proposed immunoassay according to the relationship between the current response and ROR1 concentration. Since the enzyme-linked immunosorbent assay (ELISA) reference method is not able to measure lower concentrations of ROR1, therefore it was not comparable to the designed ROR1 immunosensor. These data reveal that the fabricated immunosensor was able to detect low content of ROR1 and suitable for real sample analysis (Fig. 6). Consequently, the proposed immunosensor may afford a realistic alternative tool for the clinical diagnostics of ROR1. The obtained results were summarized in Supplementary Table S2. Based on the recorded results for CLL suffering patients, the LOD of the commercially available ELISA kits are not suitable for analyzing of ROR1 protein. The concentration of the ROR1 in the serum samples are very low, as a result, the developed immunosensor desirably detect the ROR1 concentrations. Consequently, the designed bioassay system has perfect ability to be use in clinical laboratory as a robust protocol for early stage evaluation and treatment monitoring of CLL suffering patients.

## Conclusion

A novel, label-free and cost-effective electrochemical immunosensor was fabricated for selective and ultrasensitive detection of ROR1 oncofetal antigen. Therefore, NiFe-LDH/rGO nanocomposites coated on GCE as an efficient bio-interface layer enlarge the specific surface area; enhance the conductivity and stability of the sensing platform. Also, AuNPs that attached to the modified electrode not only served as substrate materials for amplifying electron transfer, but also helps to immobilize Ab more efficiently. The offered immunosensor exhibited high performance in the detection of ROR1 with a low detection limit, wide linear range, good selectivity and long-term stability. Due to the above advantages, the resulting immunosensor can be applied as a promised tool for the detection of a wide range of cancer biomarkers.

## Supplementary Information

Supplementary Information 1.

Supplementary Information 2.
