# Supplementary material for: Ultrasensitive and label free electrochemical immunosensor for detection of ROR1 as an oncofetal biomarker using gold nanoparticles assisted LDH/rGO nanocomposite
Source: Sci Rep. 2021 Jul 21;11:14921. doi: 10.1038/s41598-021-94380-5 (PMC8295321; doi:10.1038/s41598-021-94380-5)
Supplement: Supplementary file 1 — Supplementary Information 1. [file 41598_2021_94380_MOESM1_ESM.docx]

**Supplementary Figure legends**

**Fig. S1.** The SEM images of Bare GCE, GCE/rGO, NiFe-LDH/rGO/AuNPs/ROR1sAb, NiFe-LDH/rGO/AuNPs/ROR1sAb/BSA and NiFe- LDH/rGO/AuNPs/ROR1sAb/BSA/ROR1sAg were recorded in different magnitudes (200, 500 and 1000 nm).


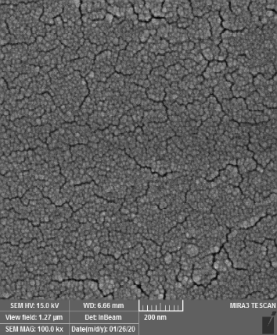


**A**


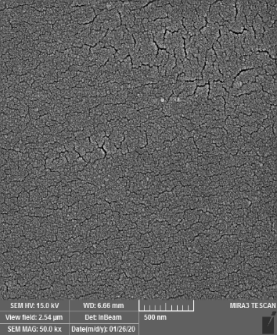


**B**


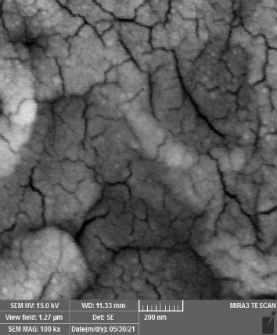


**D**


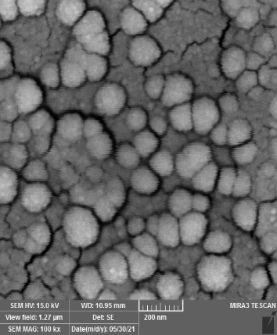


**G**


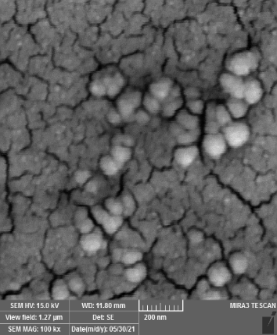


**J**


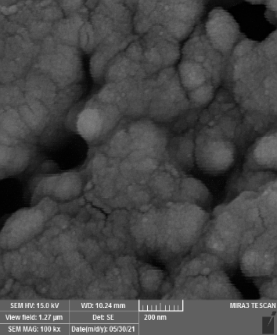


**M**


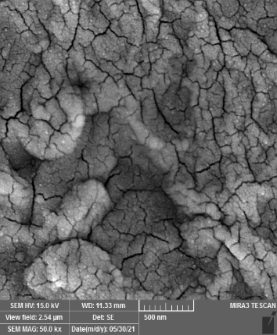


**E**


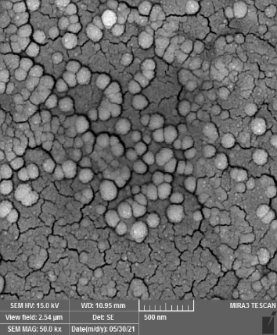


**H**


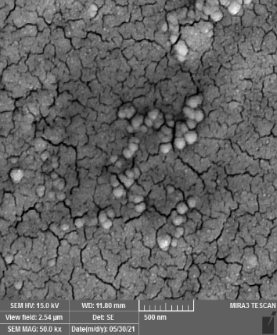


**K**


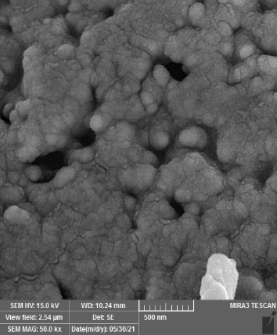


**N**


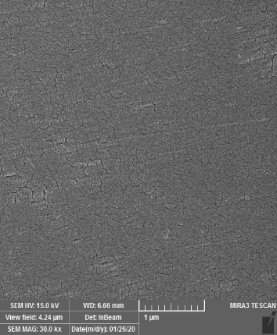


**C**


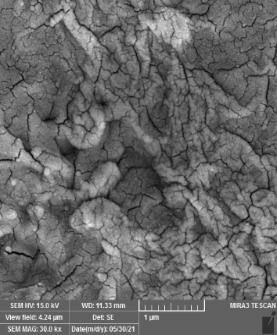


**F**


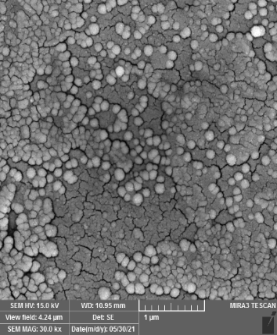


**I**


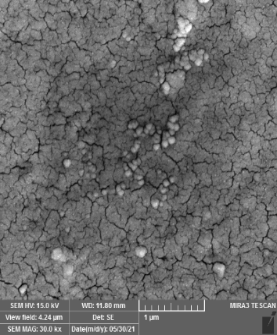


**L**


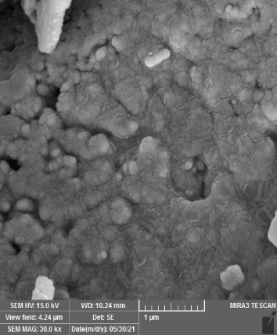


**O**
