# Supplementary material for: Ultrasensitive and label free electrochemical immunosensor for detection of ROR1 as an oncofetal biomarker using gold nanoparticles assisted LDH/rGO nanocomposite
Source: Sci Rep. 2021 Jul 21;11:14921. doi: 10.1038/s41598-021-94380-5 (PMC8295321; doi:10.1038/s41598-021-94380-5)
Supplement: Supplementary file 2 — Supplementary Information 2. [file 41598_2021_94380_MOESM2_ESM.docx]

**Supplementary table legends**

Table S1. Comparison of proposed immunosensor with Elisa immunoassay for the determination of ROR1.

| Method | Linear Range | Limit Of Detection(LOD) | References |
| --- | --- | --- | --- |
| sandwich ELISA | 25 - 250 ng/mL | 10 ng mL^-1^ | [[7](#_ENREF_7)] |
| ABCAM company's ROR1 kit | 0.819 ng/ml - 200 ng/ml | 0.082 ng mL^-1^ | [[40](#_ENREF_40)] |
| ABIN2851504 ROR1 ELISA Kit | 0.156 ng/mL - 10 ng/mL | 0.156 ng mL^-1^ | [[41](#_ENREF_41)] |
| NiFe-LDH/rGO/AuNPs immunosensor | 0.01 fg ml^-1^ - 1 pg ml^-1^ | 10 ag mL^-1^ | This work |

Table S2. Evaluation of CLL suffering patient’s serum samples with the designed immunosensor.

| Patient | Average peak height (µA) (n=5) | ROR1 concentrations (M) |
| --- | --- | --- |
| Sample A | 1.8025 | 1.73122E-16 |
| Sample B | 6.45E-01 | 2.64485E-11 |
| Sample C | 0.844284 | 3.39965E-12 |
| Sample D | 1.09E+00 | 2.56266E-13 |
| Sample E | 0.64366 | 2.69331E-11 |

Table S3. Advantages and disadvantages of the proposed electrochemical biosensor compared to conventional methods for ROR1 protein.

| Method | Advantages and disadvantages | References |
| --- | --- | --- |
| ELISA | Low sensitivity, high selectivity, Simple and fast | [[7](#_ENREF_7)] |
| Western blot | good sensitivity and specificity, Expensive, time consuming and complex | [[8](#_ENREF_8)] |
| Flow cytometry | Objective, good sensitivity, specialized training and substantial time | [[6](#_ENREF_6)] |
| NiFe-LDH/rGO/AuNPs immunosensor | High sensitivity, good selectivity, long-term stability, simple and affordable | This work |
